# Supplementary material for: HER2DX in older patients with HER2-positive early breast cancer: extended follow-up from the RESPECT trial of trastuzumab ± chemotherapy
Source: Nat Commun. 2025 Nov 4;16:9585. doi: 10.1038/s41467-025-65599-x (PMC12586486; doi:10.1038/s41467-025-65599-x)
Supplement: Supplementary file 1 — Supplementary Information [file 41467_2025_65599_MOESM1_ESM.pdf]

Supplementary Information

HER2DX in older patients with HER2-positive early breast cancer: extended follow-up from the RESPECT trial of trastuzumab ± chemotherapy

Supplementary Figure 1. CONSORT diagram of the Trans-RESPECT study. 2

Supplementary Figure 2. Relapse-free survival according to treatment arm. 2

Supplementary Figure 3. Overall survival according to treatment arm..................... 3

Supplementary Figure 4. Correlation of HER2DX IGG/immune signature score with stromal tumor-infiltrating lymphocytes (TILs). 3

Supplementary Figure 5. Relapse-free survival according to HER2DX pCR-score. 4

Supplementary Figure 6. Overall survival according to HER2DX pCR-score. 4

Supplementary Table 1. Association of clinical-pathological variables with RFS in all patients.. 5

Supplementary Table 2. Association of clinical-pathological variables with OS in all patients.. 6

Supplementary Table 3. Association of clinical-pathological variables with RFS in node-negative disease.. 7

Supplementary Table 4. Association of clinical-pathological variables with OS in node-negative disease.. 8

Supplementary Table 5. Interaction p-value of pCR score classification (High vs Med/Low) with treatment for relapse-free survival. 8

Supplementary Table 6. Interaction p-value of pCR score classification (High vs Med/Low) with treatment for OS. 9

Supplementary Table 7. Association between chemotherapy addition and overall survival by HER2DX pCR score subgroup. 9

Supplementary Figure 1. CONSORT diagram of the Trans-RESPECT study.

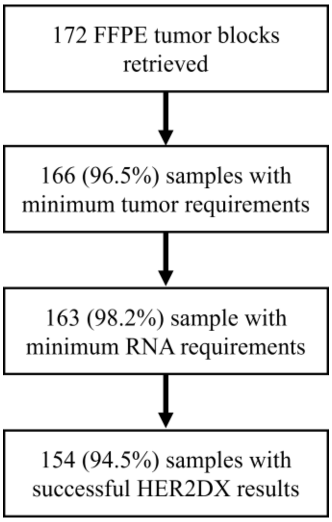

Supplementary Figure 2. Relapse-free survival according to treatment arm.

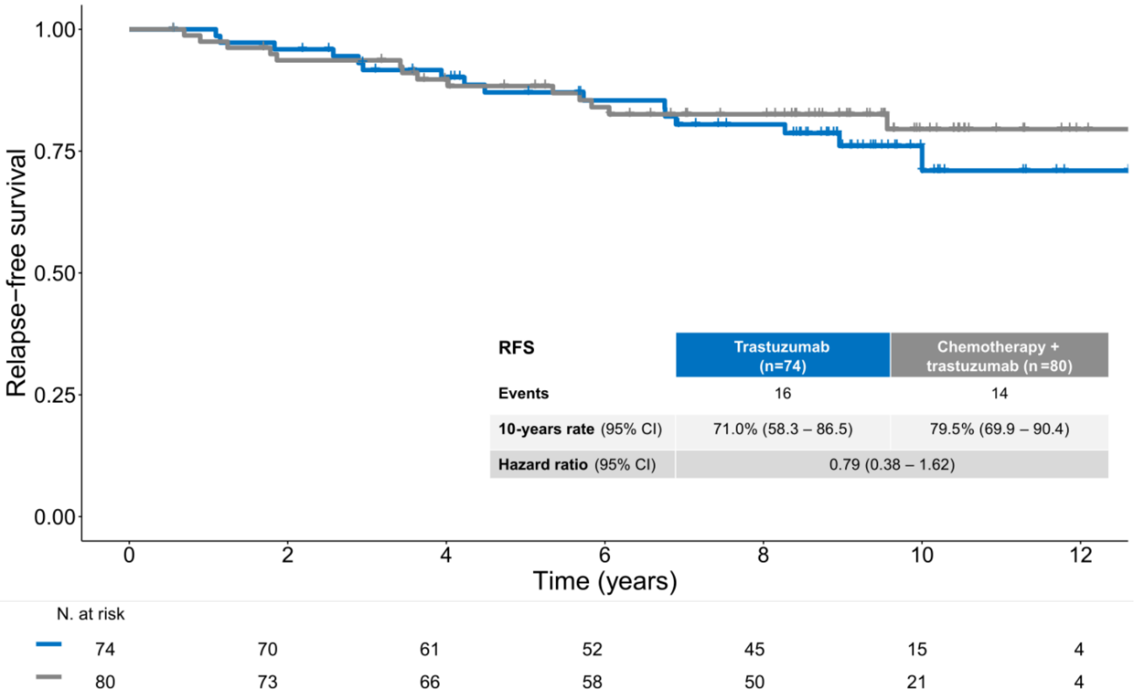

Legend: RFS: Relapse-free survival; CI: Confidence interval.

Supplementary Figure 3. Overall survival according to treatment arm.

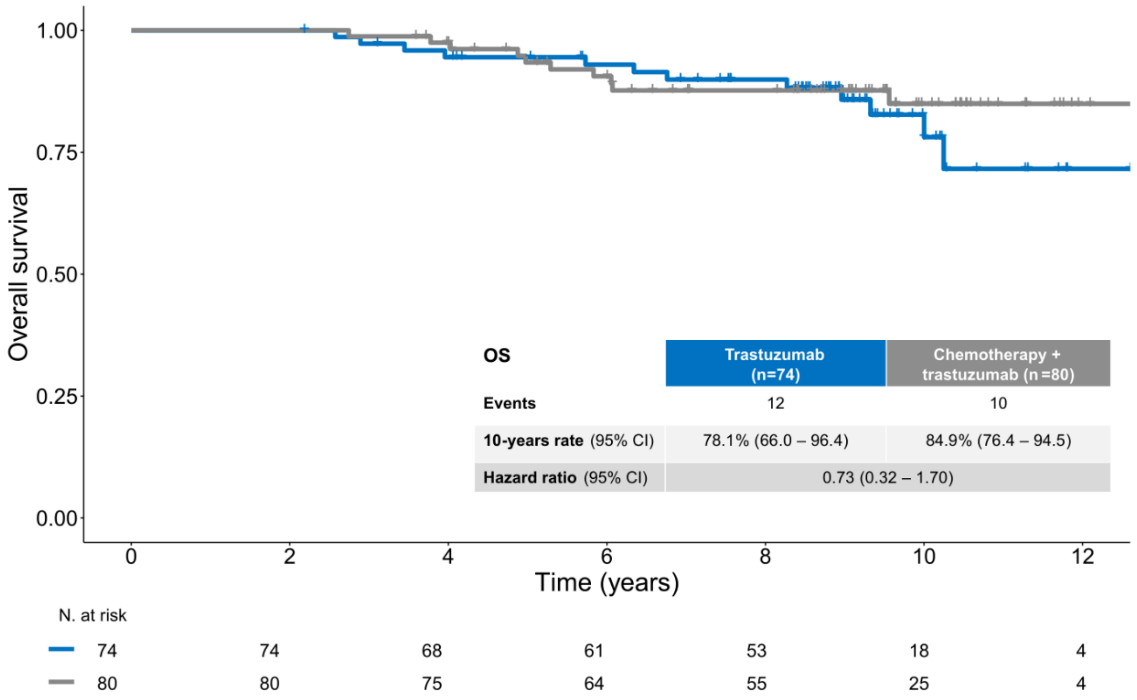

Legend: OS: Overall survival; CI: Confidence interval.

Supplementary Figure 4. Correlation of HER2DX IGG/immune signature score with stromal tumor-infiltrating lymphocytes (TILs).

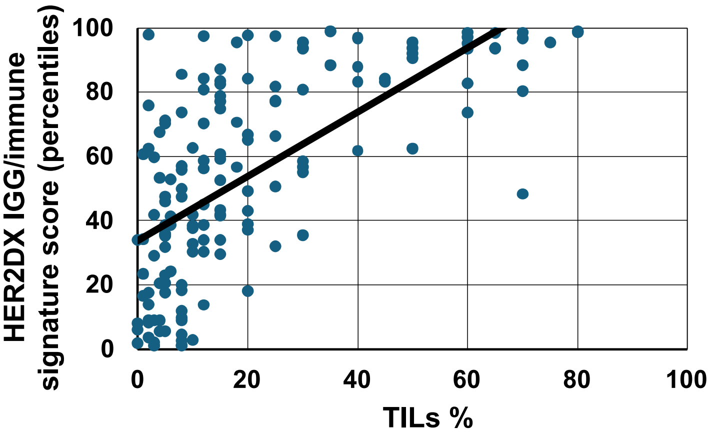

Scatter plot shows the correlation between the HER2DX IGG/immune signature score (y-axis, expressed as percentiles) and the percentage of stromal TILs (x-axis) across individual tumor samples. A positive association is observed, with a Pearson correlation coefficient of r = 0.66 and $R^2 = 0.44$, indicating that higher TIL levels are associated with higher expression of the IGG/immune gene signature included in the HER2DX assay. The black line represents the best-fit linear regression. Source data are provided as a Source Data file.

Supplementary Figure 5. Relapse-free survival according to HER2DX pCR-score.

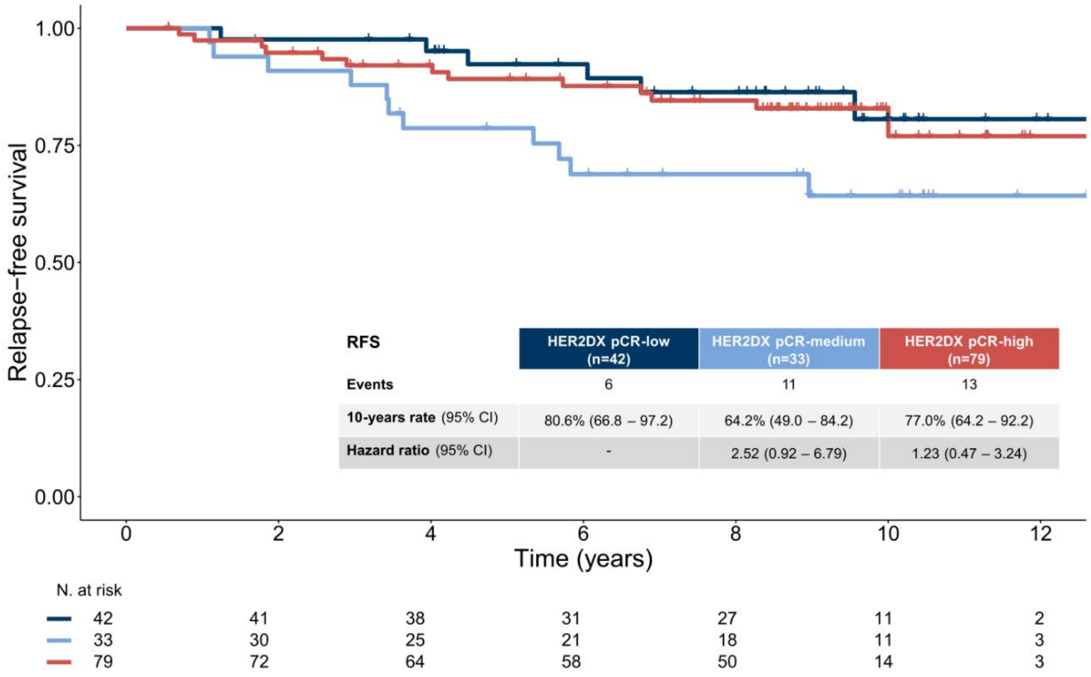

Legend: RFS: Relapse-free survival; CI: Confidence interval, pCR: Pathological complete response

Supplementary Figure 6. Overall survival according to HER2DX pCR-score.

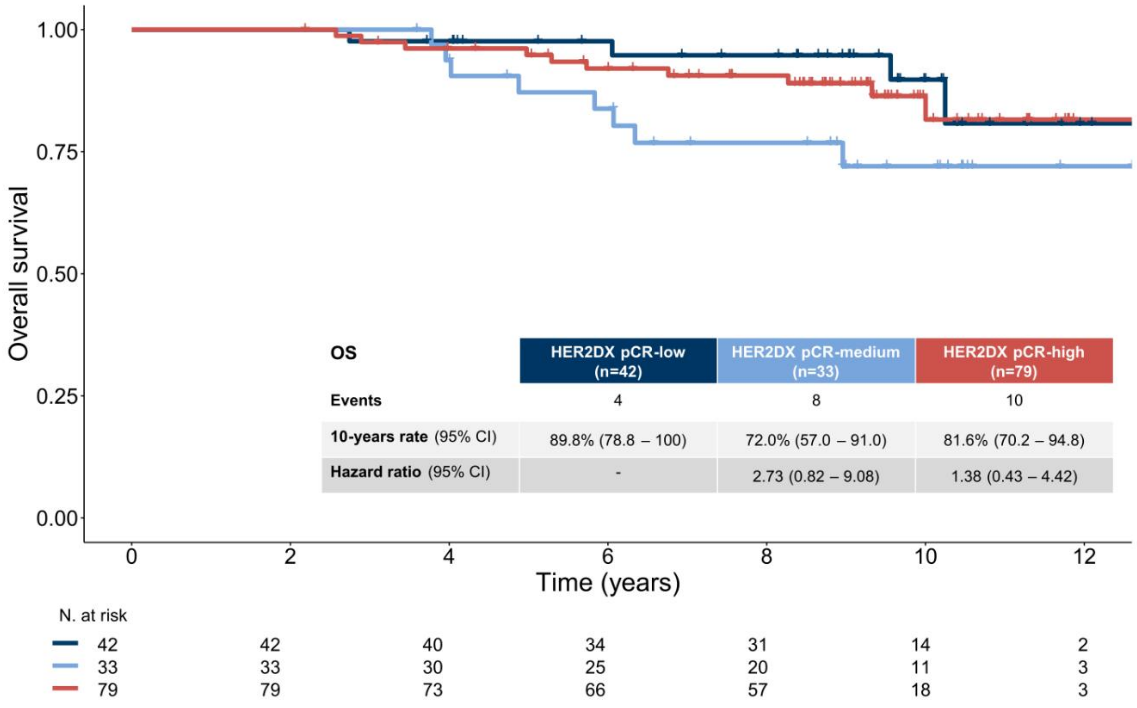

Legend: OS: Overall survival; CI: Confidence interval, pCR: Pathological complete response

Supplementary Table 1. Association of clinical-pathological variables with RFS in all patients.

Source data are provided as a Source Data file. Hazard ratios (HRs), 95% confidence intervals (CIs), and p-values were calculated using Cox proportional hazards models. All p-values are two-sided and were not adjusted for multiple comparisons.

Univariable Cox models (RFS)	HR	95% CI	P-value
**Treatment arm**			
Trastuzumab	1	-	-
Chemo+trastuzumab	0.79	0.38-1.62	0.52
**Age (continuos variable)**	1.21	1.07-1.36	0.002
**Age**			
70-73	1	-	-
74-80	2.32	1.10-4.89	0.03
**pT**			
pT1	1	-	-
pT2-3	1.81	0.87-3.77	0.11
**pN**			
pN0	1	-	-
pN1-2	1.12	0.43-2.92	0.82
**Hormone receptor status**			
Negative	1	-	-
Positive	0.68	0.32-1.42	0.30
**TILs (continuos variable)**	0.99	0.97-1.01	0.40
**TILs**			
<20%	1	-	-
≥20%	0.69	0.30-1.55	0.36

Legend: RFS: relapse-free survival, HR: hazard ratio, CI: confidence interval; pT: pathological tumor stage; pN: pathological nodal stage

Supplementary Table 2. Association of clinical-pathological variables with OS in all patients.

Source data are provided as a Source Data file. Hazard ratios (HRs), 95% confidence intervals (CIs), and p-values were calculated using Cox proportional hazards models. All p-values are two-sided and were not adjusted for multiple comparisons.

**Univariable Cox models (OS)**	**HR**	**95% CI**	**P-value**
**Treatment arm**			
Trastuzumab	1	-	-
Chemo+trastuzumab	0.73	0.32-1.70	0.47
**Age (continuos variable)**	1.26	1.09-1.46	0.002
**Age**			
70-73	1	-	-
74-80	3.12	1.27-7.68	0.01
**pT**			
pT1	1	-	-
pT2-3	2.19	0.92-5.23	0.08
**pN**			
pN0	1	-	-
pN1-2	0.90	0.27-3.06	0.87
**Hormone receptor status**			
Negative	1	-	-
Positive	0.46	0.18-1.18	0.11
**TILs (continuos variable)**	0.99	0.96-1.01	0.35
**TILs**			
<20%	1	-	-
≥20%	0.68	0.26-1.74	0.42

Legend: OS: overall survival, HR: hazard ratio, CI: confidence interval; pT: pathological tumor stage; pN: pathological nodal stage

Supplementary Table 3. Association of clinical-pathological variables with RFS in node-negative disease.

Source data are provided as a Source Data file. Hazard ratios (HRs), 95% confidence intervals (CIs), and p-values were calculated using Cox proportional hazards models. All p-values are two-sided and were not adjusted for multiple comparisons.

**Univariable Cox models (RFS, node-negative)**	**HR**	**95% CI**	**P-value**
**Treatment arm**			
Trastuzumab	1	-	-
Chemo+trastuzumab	0.52	0.22-1.21	0.13
**Age (continuos variable)**	1.23	1.07-1.41	0.004
**Age**			
70-73	1	-	-
74-80	2.63	1.15-6.02	0.02
**pT**			
pT1	1	-	-
pT2-3	1.56	0.70-3.49	0.28
**pN**			
pN0	-	-	-
pN1-2	-	-	-
**Hormone receptor status**			
Negative	1	-	-
Positive	0.71	0.31-1.61	0.41
**TILs (continuos variable)**	0.99	0.97-1.02	0.55
**TILs**			
<20%	1	-	-
≥20%	0.67	0.26-1.69	0.39

Legend: RFS: relapse-free survival, HR: hazard ratio, CI: confidence interval; pT: pathological tumor stage; pN: pathological nodal stage

Supplementary Table 4. Association of clinical-pathological variables with OS in node-negative disease.

Source data are provided as a Source Data file. Hazard ratios (HRs), 95% confidence intervals (CIs), and p-values were calculated using Cox proportional hazards models. All p-values are two-sided and were not adjusted for multiple comparisons.

Univariable Cox models (OS, node-negative)	HR	95% CI	P-value
**Treatment arm**			
Trastuzumab	1	-	-
Chemo+trastuzumab	0.50	0.19-1.33	0.17
**Age (continuos variable)**	1.28	1.08-1.50	0.003
**Age**			
70-73	1	-	-
74-80	3.41	1.27-9.13	0.02
**pT**			
pT1	1	-	-
pT2-3	2.13	0.82-5.50	0.12
**pN**			
pN0	-	-	-
pN1-2	-	-	-
**Hormone receptor status**			
Negative	1	-	-
Positive	0.48	0.17-1.36	0.17
**TILs (continuos variable)**	0.99	0.97-1.02	0.52
**TILs**			
<20%	1	-	-
≥20%	0.74	0.26-2.10	0.57

Legend: OS: overall survival, HR: hazard ratio, CI: confidence interval; pT: pathological tumor stage; pN: pathological nodal stage

Supplementary Table 5. Interaction p-value of pCR score classification (High vs Med/Low) with treatment for relapse-free survival.

Source data are provided as a Source Data file. Interaction biomarker-outcome p-values were calculated using Cox proportional hazards models. All p-values are two-sided and were not adjusted for multiple comparisons.

RFS Univariate Analysis	Interaction P-value
**No censoring**	0.200
**Censoring at 10yrs**	0.272
**Censoring at 9yrs**	0.358
**Censoring at 8yrs**	0.358
**Censoring at 7yrs**	0.358
**Censoring at 6yrs**	0.646

**Censoring at 5yrs**	0.741

Supplementary Table 6. Interaction p-value of pCR score classification (High vs Med/Low) with treatment for OS.

Source data are provided as a Source Data file. Interaction biomarker-outcome p-values were calculated using Cox proportional hazards models. All p-values are two-sided and were not adjusted for multiple comparisons.

**OS Univariate Analysis**	**Interaction P-value**
**No censoring**	0.045
**Censoring at 10yrs**	0.039
**Censoring at 9yrs**	0.088
**Censoring at 8yrs**	0.081
**Censoring at 7yrs**	0.081
**Censoring at 6yrs**	0.127
**Censoring at 5yrs**	0.151

Supplementary Table 7. Association between chemotherapy addition and overall survival by HER2DX pCR score subgroup.

Source data are provided as a Source Data file.

**Variable**	**Low**	**Medium**	**High**
N	42	33	79
Hazard Ratio (Chemotherapy vs no chemotherapy)	2.12	1.37	0.23
95% CI (lower)	0.21	0.33	0.05
95% CI (upper)	21.25	5.75	1.08
P-value (log-rank test)	0.5	0.7	0.04
