# Supplementary material for: HER2DX in older patients with HER2-positive early breast cancer: extended follow-up from the RESPECT trial of trastuzumab ± chemotherapy
Source: Nat Commun. 2025 Nov 4;16:9585. doi: 10.1038/s41467-025-65599-x (PMC12586486; doi:10.1038/s41467-025-65599-x)
Supplement: Supplementary file 3 — Reporting Summary [file 41467_2025_65599_MOESM3_ESM.pdf]

Corresponding author(s): Aleix Prat

Last updated by author(s): 13th Septemeber 2025

Reporting Summary

Nature Portfolio wishes to improve the reproducibility of the work that we publish. This form provides structure for consistency and transparency in reporting. For further information on Nature Portfolio policies, see our Editorial Policies and the Editorial Policy Checklist.

Statistics

For all statistical analyses, confirm that the following items are present in the figure legend, table legend, main text, or Methods section.

n/a	Confirmed	
☐	☒	The exact sample size (*n*) for each experimental group/condition, given as a discrete number and unit of measurement
☐	☒	A statement on whether measurements were taken from distinct samples or whether the same sample was measured repeatedly
☐	☒	The statistical test(s) used AND whether they are one- or two-sided<br>*Only common tests should be described solely by name; describe more complex techniques in the Methods section.*
☐	☒	A description of all covariates tested
☐	☒	A description of any assumptions or corrections, such as tests of normality and adjustment for multiple comparisons
☐	☒	A full description of the statistical parameters including central tendency (e.g. means) or other basic estimates (e.g. regression coefficient) AND variation (e.g. standard deviation) or associated estimates of uncertainty (e.g. confidence intervals)
☐	☒	For null hypothesis testing, the test statistic (e.g. F, t, r) with confidence intervals, effect sizes, degrees of freedom and P value noted<br>*Give P values as exact values whenever suitable.*
☒	☐	For Bayesian analysis, information on the choice of priors and Markov chain Monte Carlo settings
☒	☐	For hierarchical and complex designs, identification of the appropriate level for tests and full reporting of outcomes
☐	☒	Estimates of effect sizes (e.g. Cohen's *d*, Pearson's *r*), indicating how they were calculated

Our web collection on statistics for biologists contains articles on many of the points above.

Software and code

Policy information about availability of computer code

Data collection	Gene expression data were processed through a custom pipeline developed by Reveal Genomics. This proprietary software performs quality control, normalization, and raw-count aggregation on NanoString nCounter output files. The source code is an industrial trade secret and cannot be publicly released. Qualified investigators may request access under a confidentiality and licensing agreement by contacting Reveal Genomics (data-access@revealgenomics.com).
Data analysis	The HER2DX assay comprises four weighted gene signatures whose composition (i.e., which genes are included and the direction of their contribution to pCR and survival endpoints) is fully described in Prat et al., EBioMedicine 2020. However, the numeric coefficients that link each signature to the clinical endpoints are proprietary to Reveal Genomics and cannot be disclosed. Academic groups wishing to validate or benchmark the HER2DX algorithm may request a license and access to the coefficient values under a data- and code-transfer agreement, subject to prior Ethics Committee approval.

For manuscripts utilizing custom algorithms or software that are central to the research but not yet described in published literature, software must be made available to editors and reviewers. We strongly encourage code deposition in a community repository (e.g. GitHub). See the Nature Portfolio guidelines for submitting code & software for further information.

Data

Policy information about availability of data

All manuscripts must include a data availability statement. This statement should provide the following information, where applicable:

- Accession codes, unique identifiers, or web links for publicly available datasets
- A description of any restrictions on data availability
- For clinical datasets or third party data, please ensure that the statement adheres to our policy

Source data underlying all figures and tables are provided with this paper as the Source Data file. The protocol of the RESPECT study is provided as Supplementary Data. Individual-level clinical and genomic data generated for this study (including RNA expression values used to derive HER2DX variables and clinical outcomes) are available under restricted access because participant consent does not permit open public posting and the HER2DX assay is subject to intellectual-property and licensing constraints. Access may be granted to qualified academic investigators for non-commercial research upon approval by the data custodians and execution of a data transfer agreement. Requests should include a brief research proposal and documentation of local ethics approval and be sent to alprat@clinic.cat. Requests will receive an initial response within 30 business days; if approved, de-identified datasets will be provided via secure transfer within 30 business days of agreement execution. Data will be available for a minimum of 5 years from the date of publication. Sharing is subject to standard restrictions prohibiting re-identification, re-distribution, and commercial use.

Research involving human participants, their data, or biological material

Policy information about studies with human participants or human data. See also policy information about sex, gender (identity/presentation), and sexual orientation and race, ethnicity and racism.

Reporting on sex and gender	The RESPECT trial (JCO 20.00184) was carried out exclusively in Japan and enrolled only female patients.Sex and gender reporting. Patient sex (female) was abstracted from medical records; no information on gender identity was collected or is available. Consequently, gender-based analyses are not applicable.
Reporting on race, ethnicity, or other socially relevant groupings	No formal race or ethnicity data were collected. All participants were Japanese women enrolled at 99 oncology centers across Japan, and self-identified as of Japanese nationality and ethnicity. No further categorization was performed, and race/ethnicity was not used as a covariate or proxy for other social factors.
Population characteristics	Eligible patients were women aged ≥ 70 and < 81 years with histologically confirmed invasive, HER2-positive (IHC 3+ or FISH+) breast cancer, stage I (pT ≥ 1 cm), IIA, IIB, or IIIA (M0). Key baseline covariates included ECOG performance status (0–1), lymph node status (present/none), hormone-receptor status, and cardiac function (LVEF ≥ 55% within 4 weeks prior to registration). Median age and full demographics are summarized in Table 1 of the main manuscript. https://ascopubs.org/doi/10.1200/JCO.20.00184
Recruitment	From October 2009 through November 2014, a total of 275 patients aged 70-80 years with HER2-positive invasive breast cancer were enrolled from 99 institutions under the CSPOR umbrella. Patients were identified from routine post-surgical follow-up and registered via faxed case forms to the CSPOR Data Center, which performed central eligibility checks and dynamic allocation (minimization)
Ethics oversight	All individual patient data used in this study were accessed under a formal data-sharing agreement between the CSPOR and Reveal Genomics. Data were pseudonymized at the source in accordance with applicable data-protection regulations. The translational study was approved by the CSPOR Review Board; internal governance approval was granted by the Reveal Genomics Executive Board. All procedures conformed to institutional and international ethical standards, including the Declaration of Helsinki. Written informed consent was obtained from RESPECT trial participants.

Note that full information on the approval of the study protocol must also be provided in the manuscript.

Field-specific reporting

Please select the one below that is the best fit for your research. If you are not sure, read the appropriate sections before making your selection.

☒ Life sciences ☐ Behavioural & social sciences ☐ Ecological, evolutionary & environmental sciences

For a reference copy of the document with all sections, see nature.com/documents/nr-reporting-summary-flat.pdf

Life sciences study design

All studies must disclose on these points even when the disclosure is negative.

Sample size	The original trial recruited a total of 275 patients aged 70-80 years with HER2-positive invasive breast cancer were enrolled from 99 institutions. Nine patients (3.3%) were excluded, leaving 266 for full-set analysis (trastuzumab monotherapy [n = 135] and trastuzumab + chemotherapy [n = 131]). For the HER2DX analysis on this trial, we used all available tumor samples from the trial. The final sample size was 154 out of 266 eligible patients (58.0%) from the RESPECT trial. This subset reflects the 160 tumor blocks retrieved from local sites who agreed to participate in the translational study, of which 96.3% were successfully profiled, as shown in Figure S1.
Data exclusions	No data exclusions were performed in the 156 HER2DX sample set.

Replication	No replication of the test was performed. The HER2DX genomic test is performed once, per each tumor sample. This is a standarized test under CLIA/CAP accreditation in the US, and undergoing CE mark in Europe.
Randomization	A 1:1 randomization was performed in the original trial (Sawaki et al. JCO 2020). The original trial has been previously published: https://ascopubs.org/doi/10.1200/JCO.20.00184. Randomization was performed at the data center after confirming patient eligibility with assignment adjustment factors, as follows: age (70-75 v 76-80 years), performance status (0 v 1), hormone receptor status (positive [≥ 10%] v negative), pathologic nodal status (positive v negative), and participating institution.
Blinding	The HER2DX genomic test was performed in the central lab in Barcelona, Spain, blinded from clinical data.

Reporting for specific materials, systems and methods

We require information from authors about some types of materials, experimental systems and methods used in many studies. Here, indicate whether each material, system or method listed is relevant to your study. If you are not sure if a list item applies to your research, read the appropriate section before selecting a response.

Materials & experimental systems

n/a	Involved in the study
☐	☐ Antibodies
☐	☐ Eukaryotic cell lines
☐	☐ Palaeontology and archaeology
☐	☐ Animals and other organisms
☐	☒ Clinical data
☐	☐ Dual use research of concern
☐	☐ Plants

Methods

n/a	Involved in the study
☐	☐ ChIP-seq
☐	☐ Flow cytometry
☐	☐ MRI-based neuroimaging

Antibodies

Antibodies used	N/A
Validation	N/A

Eukaryotic cell lines

Policy information about cell lines and Sex and Gender in Research

Cell line source(s)	N/A
Authentication	N/A
Mycoplasma contamination	N/A
Commonly misidentified lines (See ICLAC register)	N/A

Palaeontology and Archaeology

Specimen provenance	N/A
Specimen deposition	N/A
Dating methods	N/A

☐ Tick this box to confirm that the raw and calibrated dates are available in the paper or in Supplementary Information.

Ethics oversight	N/A

Note that full information on the approval of the study protocol must also be provided in the manuscript.

Animals and other research organisms

Policy information about studies involving animals; ARRIVE guidelines recommended for reporting animal research, and Sex and Gender in Research

Laboratory animals	N/A
Wild animals	N/A
Reporting on sex	N/A
Field-collected samples	N/A
Ethics oversight	N/A

Note that full information on the approval of the study protocol must also be provided in the manuscript.

Clinical data

Policy information about clinical studies

All manuscripts should comply with the ICMJE guidelines for publication of clinical research and a completed CONSORT checklist must be included with all submissions.

Clinical trial registration	NCT01104935
Study protocol	https://ascopubs.org/action/downloadSupplement?doi=10.1200%2FJCO.20.00184&file=Protocol_jco.20.00184.pdf
Data collection	From October 2009 through November 2014, a total of 275 patients aged 70-80 years with HER2-positive invasive breast cancer were enrolled from 99 institutions.
Outcomes	The co-primary endpoints were RFS, defined as the time from randomization to the first invasive recurrence, second primary cancer, or death from any cause, and OS, defined as the time from randomization to death from any cause.

Dual use research of concern

Policy information about dual use research of concern

Hazards

Could the accidental, deliberate or reckless misuse of agents or technologies generated in the work, or the application of information presented in the manuscript, pose a threat to:

No	Yes	
☒	☐	Public health
☒	☐	National security
☒	☐	Crops and/or livestock
☒	☐	Ecosystems
☒	☐	Any other significant area

Experiments of concern

Does the work involve any of these experiments of concern:

No	Yes	
☒	☐	Demonstrate how to render a vaccine ineffective
☒	☐	Confer resistance to therapeutically useful antibiotics or antiviral agents
☒	☐	Enhance the virulence of a pathogen or render a nonpathogen virulent
☒	☐	Increase transmissibility of a pathogen
☒	☐	Alter the host range of a pathogen
☒	☐	Enable evasion of diagnostic/detection modalities
☒	☐	Enable the weaponization of a biological agent or toxin
☒	☐	Any other potentially harmful combination of experiments and agents

Plants

Seed stocks	N/A
Novel plant genotypes	N/A
Authentication	N/A

ChIP-seq

Data deposition

- [ ] Confirm that both raw and final processed data have been deposited in a public database such as GEO.
- [ ] Confirm that you have deposited or provided access to graph files (e.g. BED files) for the called peaks.

Data access links<br>*May remain private before publication.*	N/A
Files in database submission	N/A
Genome browser session<br>(e.g. UCSC)	N/A

Methodology

Replicates	N/A
Sequencing depth	N/A
Antibodies	N/A
Peak calling parameters	N/A
Data quality	N/A
Software	N/A

Flow Cytometry

Plots

Confirm that:

- [ ] The axis labels state the marker and fluorochrome used (e.g. CD4-FITC).
- [ ] The axis scales are clearly visible. Include numbers along axes only for bottom left plot of group (a 'group' is an analysis of identical markers).
- [ ] All plots are contour plots with outliers or pseudocolor plots.
- [ ] A numerical value for number of cells or percentage (with statistics) is provided.

Methodology

Sample preparation	N/A
Instrument	N/A
Software	N/A
Cell population abundance	N/A

Gating strategy | N/A

☐ Tick this box to confirm that a figure exemplifying the gating strategy is provided in the Supplementary Information.

Magnetic resonance imaging

Experimental design

Design type | N/A

Design specifications | N/A

Behavioral performance measures | N/A

Acquisition

Imaging type(s) | N/A

Field strength | N/A

Sequence & imaging parameters | N/A

Area of acquisition | N/A

Diffusion MRI ☐ Used ☒ Not used

Preprocessing

Preprocessing software | N/A

Normalization | N/A

Normalization template | N/A

Noise and artifact removal | N/A

Volume censoring | N/A

Statistical modeling & inference

Model type and settings | N/A

Effect(s) tested | N/A

Specify type of analysis: ☐ Whole brain ☐ ROI-based ☐ Both

Statistic type for inference (See Eklund et al. 2016) | N/A

Correction | N/A

Models & analysis

n/a	Involved in the study
☒	☐ Functional and/or effective connectivity
☒	☐ Graph analysis
☒	☐ Multivariate modeling or predictive analysis
